# An association mapping approach to identify favourable alleles for tomato fruit quality breeding

**DOI:** 10.1186/s12870-014-0337-9

**Published:** 2014-12-03

**Authors:** Valentino Ruggieri, Gianluca Francese, Adriana Sacco, Antonietta D’Alessandro, Maria Manuela Rigano, Mario Parisi, Marco Milone, Teodoro Cardi, Giuseppe Mennella, Amalia Barone

**Affiliations:** Department of Agricultural Sciences, University of Naples Federico II, Via Università 100, 80055 Portici, Italy; Consiglio per la Ricerca e la Sperimentazione in Agricoltura - Centro di Ricerca per l’Orticoltura (CRA-ORT), Via Cavalleggeri 25, 84098 Pontecagnano, SA Italy

**Keywords:** Candidate genes, Fruit quality, Genome-wide association, Metabolite analysis, Mixed Linear Model, *Solanum lycopersicum*, SolCAP Infinium array

## Abstract

**Background:**

Genome Wide Association Studies (GWAS) have been recently used to dissect complex quantitative traits and identify candidate genes affecting phenotype variation of polygenic traits. In order to map loci controlling variation in tomato marketable and nutritional fruit traits, we used a collection of 96 cultivated genotypes, including Italian, Latin American, and other worldwide-spread landraces and varieties. Phenotyping was carried out by measuring ten quality traits and metabolites in red ripe fruits. In parallel, genotyping was carried out by using the Illumina Infinium SolCAP array, which allows data to be collected from 7,720 single nucleotide polymorphism (SNP) markers.

**Results:**

The Mixed Linear Model used to detect associations between markers and traits allowed population structure and relatedness to be evidenced within our collection, which have been taken into consideration for association analysis. GWAS identified 20 SNPs that were significantly associated with seven out of ten traits considered. In particular, our analysis revealed two markers associated with phenolic compounds, three with ascorbic acid, β-carotene and *trans*-lycopene, six with titratable acidity, and only one with pH and fresh weight. Co-localization of a group of associated loci with candidate genes/QTLs previously reported in other studies validated the approach. Moreover, 19 putative genes in linkage disequilibrium with markers were found. These genes might be involved in the biosynthetic pathways of the traits analyzed or might be implied in their transcriptional regulation. Finally, favourable allelic combinations between associated loci were identified that could be pyramided to obtain new improved genotypes.

**Conclusions:**

Our results led to the identification of promising candidate loci controlling fruit quality that, in the future, might be transferred into tomato genotypes by Marker Assisted Selection or genetic engineering, and highlighted that intraspecific variability might be still exploited for enhancing tomato fruit quality.

**Electronic supplementary material:**

The online version of this article (doi:10.1186/s12870-014-0337-9) contains supplementary material, which is available to authorized users.

## Background

The genetic architecture of nutritional and quality traits in tomato has been extensively investigated due to the economic importance of this species worldwide. However, the genetic dissection of such traits is a challenging task due to their quantitative inheritance. To assist in this effort, an increasing number of genomic and genetic resources are today exploitable, including genome and transcriptome sequences, dense SNP maps, germplasm collections and public databases of genomic information [1-6]. The availability of these resources, the recent advances in high-throughput genomic platforms and the increasing interest in exploring natural genetic diversity, make association mapping an appealing and affordable approach to identify genes responsible for quantitative variation of complex traits. In the recent years, in order to dissect complex quantitative traits and identify candidate genes affecting such traits, the association mapping approach has been widely used [7-10]. This strategy relies on detecting linkage disequilibrium (LD) between genetic markers and genes controlling the phenotype of interest by exploiting the recombination events accumulating over many generations and thus increasing the accuracy of the associations detected. It offers several advantages over traditional linkage mapping, including an increased resolution, a reduced research time and a higher allele number detection [9,11]. In addition, genome-wide association studies (GWAS) make it possible to simultaneously screen a large number of accessions for genetic variation, thus allowing identification of novel and superior alleles underlying diverse complex traits [12].

Many association studies have been published to date for studying morpho-physical and fruit quality traits in tomato. Mazzucato *et al*. [13] studied associations for 15 morpho-physiological traits using 29 Simple Sequence Repeat (SSR) markers in a collection of 61 accessions including mainly Italian tomato landraces. Recently, Ranc *et al*. [14] and Xu *et al*. [15] investigated morphological and fruit quality traits in cultivated tomato and its related wild species by using 352 and 192 markers, respectively. Shirasawa *et al*. [16] studied the association with agronomical traits, such as fruit size, shape and plant architecture, using an Illumina GoldenGate assay for 1,536 SNPs.

Association mapping requires high-density oligonucleotide arrays to efficiently identify SNPs distributed across the genome at a density that accurately reflects genome-wide LD structure and haplotype diversity. For tomato, a high-density single nucleotide polymorphism (SNP) array was recently built, which resulted suitable for genome-wide association analysis. The SolCAP array, with 7,720 SNPs based on polymorphic transcriptome sequences from six tomato accessions [2], is actually the largest platform to genotype tomato collections. The SNP distribution on the array reflects their origin, since they mostly derive from ESTs and thus from the euchromatic genomic regions, which in tomato have a very typical sub-telomeric distribution. The SolCAP platform was recently used to infer SNP effects on gene functions in tomato [17], to map two suppressors of OVATE (*ov*) loci [18], to reveal detailed representation of the molecular variation and structure of *S. lycopersicum* [19], to investigate the effect of contemporary breeding on the tomato genome [5] and to identify candidate loci for fruit metabolic traits [20]. Here, a genome-wide association study in a collection of 96 tomato genotypes was undertaken using this high-quality custom-designed genotyping array. Phenotypic data for ten nutritional and quality traits were recorded over two consecutive field seasons. Using this strategy, additional associations and putative novel candidate genes were detected, compared to previous association studies that were carried out for some of the traits analysed in this study [14,15,20,21].

## Results

### Phenotyping

The tomato collection was phenotyped for five nutritional and five fruit quality traits. The former group included metabolites with antioxidant activity, such as ascorbic acid (AsA), β-carotene (β-C) *cis*-lycopene (*c-*LYC*)*, *trans*-lycopene (*t*-LYC) and phenolics (PHE), whereas the latter consisted of dry matter (DMW) and fresh fruit weight (FW), pH, soluble solids content (SSC) and titratable acidity (TA). Detailed information on phenotyping performed for each trait and genotype is reported in Additional file 1.

**Figure 1:**
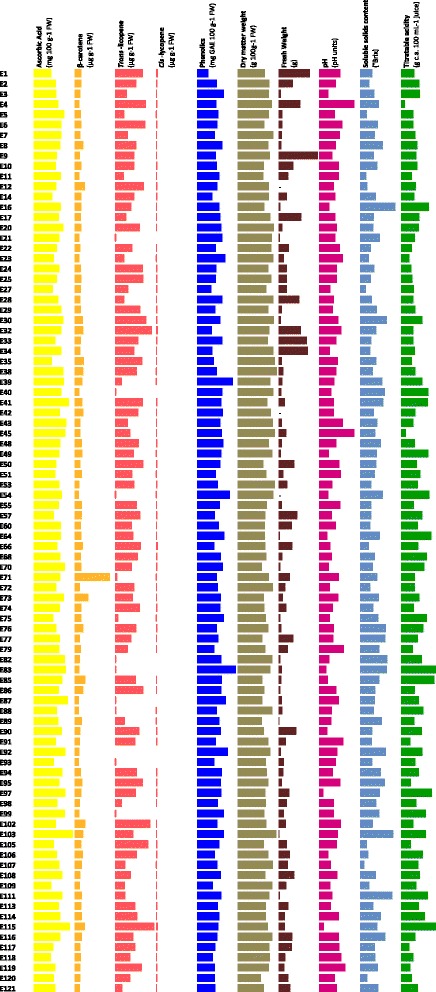
**Trend of variation of nutritional and quality traits in the tomato collection.** Each bar represents the mean of two years values.

Heritability values calculated on the two years of phenotypic characterization were higher than 0.5 for all traits except than for *cis*-lycopene (Table 1). Therefore, phenotypes data were averaged over the two years, and the minimum, maximum and mean values are reported in Table 1, together with the coefficient of variation (% CV). A large range of variation was found for all traits, as also shown in Figure 1. In particular, in the figure is clearly evident that for β-carotene the genotype E71 represents an outlier, since it exhibited a value of 25 μg g^−1^ FW compared to 1.99 μg g^−1^ FW mean value of the whole population. Indeed, the genotype E71 corresponds to the variety Caro Red, which was specifically selected for this trait [22]. Consequently, in order to prevent bias, the genotype E71 was excluded from subsequent analyses. As for the other traits, variability estimated by the coefficient of variation ranged from values of approximately 10% to 50%, with only one trait (pH) showing a very low CV value (2.87) and one trait (FW) exhibiting a very high CV value (90.9%).

**Table 1:** **Phenotypic variation of traits analysed in the whole collection**

**Trait**	***H*** ^**2**^	**Min**	**Max**	**Mean**	**CV%**
Ascorbic Acid (mg 100 g^−1^ FW)	0.56	22.40	51.23	33.59	17.33
β-carotene (μg g^−1^ FW)^a^	0.75	0.11	7.79	1.99	60.02
*Trans*-lycopene (μg g^−1^ FW)	0.61	0.19	193.17	85.50	54.97
*Cis*-lycopene (μg g^−1^ FW)	0.48	0.00	8.60	3.40	56.13
Phenolics (mg GAE 100 g^−1^ FW)	0.52	25.06	86.23	48.73	20.47
Dry matter weight (g 100 g^−1^ FW)	0.53	6.50	10.93	8.56	12.47
Fresh Weight (g)	0.87	5.35	313.50	65.92	90.92
pH (pH units)	0.64	4.08	4.79	4.35	2.87
Soluble solids content (Brix)	0.87	5.13	8.90	6.60	11.54
Titratable acidity (g c.a. 100 mL^−1^ juice)	0.66	0.27	0.75	0.47	21.54

Heritability (*H* ^2^), minimum (min), maximum (max) and mean values, and coefficient of variation (CV%) are shown for each trait.

^a^All data reported for β-carotene are referred to the whole collection except than genotype E71 (see Figure 1).

The Pearson correlation coefficients (*r*) among traits (Additional file 2) showed a positive value between *t*-LYC and *c*-LYC (*r* = 0.89) and a negative value between pH and TA (*r* = −0.70). In addition, AsA, PHE and TA were positively correlated with SSC and negatively correlated with FW. PHE content was also negatively correlated with *t-LYC* and *c-*LYC (*r* = −0.38 and −0.49, respectively), whereas it was positively correlated with AsA (*r* = 0.55).

### Genotyping and population structure

Genotyping was performed using the Illumina array consisting of 7,720 bi-allelic SNPs. On average, there were 638 SNPs per chromosome with a minimum number for chromosome 12 (391 SNPs) and a maximum for chromosome 11 (1,061 SNPs). Eighty-one SNPs with missing data >10% were removed from the dataset. Of the remaining 7,639 SNPs, 2,072 (27% of total SNPs) were monomorphic, 2,626 (34%) were polymorphic with MAF < 5% and finally 2,941 (38.4%) were polymorphic with MAF >5%. On removing SNPs with MAF <5% the average number per chromosome decreased to 241. The minimum value was detected for chromosome 10 and the maximum for chromosome 11. The distribution of total SNPs and of SNPs with MAF > 5% across chromosomes is summarized in Additional file 3. The extent of LD across each chromosome was also estimated. Pairwise *r*^2^ was calculated using 2,941 polymorphic SNPs with MAF > 5%. The *r*^2^ values were plotted against the genetic distance, and curves of LD decay were fitted using the LOWESS algorithm. The average extent of LD across each chromosome was thus estimated based on the intersections of the LOWESS curves with LD significance baselines and among three different critical values considered (0.2, 0.3 and 0.5) a 0.2 baseline was used to predict the highest reliable decay, following also previous results reported in tomato [5]. The distance of LD decay ranged from 1,968 kbp for chromosome 11 to 287 kbp for chromosome 2 and an average value of 665 kbp was found (Additional files 4 and 5). According to LD decay values, we selected a subset of 600 potentially unlinked SNPs for inferring population structure. The model used indicated K = 3 as the best number of sub-populations (hereafter referred to as Q = 3), providing support for the existence of three distinct clusters in our association panel. STRUCTURE results and Delta K plot are graphed in Additional file 6. A multiple regression analysis was run to predict the effect of population structure on the analysed traits (Table 2). No effect was statistically predictable for three traits, whereas a low/moderate effect was detected for β-C (R^2^ = 7.3%), *c-*Lyc (R^2^ = 10.5%), DMW (R^2^ = 10.8%), SSC (R^2^ = 11.5%), AsA (R^2^ = 17%) and PHE (R^2^ = 17.5%). A greater effect was observed for FW, since more than 40% of phenotypic variance was explained by the population structure. The relative kinship was also estimated and the matrix of genetic relatedness is presented as a heat map in Additional file 7. By using the set of markers with MAF > 5% more than 60% of the pairwise kinship estimates ranged from 1 to 1.5 (on a scale from 0 to 2), 16% from 0.5 to 1 and only 10% ranged from 0 to 0.5., whereas by using MAF > 10%, 47%, 39% and 12% of the pairwise estimates ranged from 1 to 1.5, from 0.5 to 1 and from 0 to 0.5, respectively.

**Table 2:** **Multiple regression analysis between phenotypic traits and population structure**

**Traits**	**Regression results**	
	**R** ^**2**^	**P-value**
Ascorbic Acid	0.170	0.001
β-carotene	0.073	0.050
*Trans*-lycopene	0.053	0.118
*Cis*-lycopene	0.105	0.013
Phenolics	0.175	0.001
Dry matter weight	0.108	0.011
Fresh Weight	0.416	0.001
pH	0.006	0.780
Soluble solids content	0.115	0.008
Titratable acidity	0.038	0.219

Proportion of variance accounted for by population structure (R^2^) and statistical significance of the model (P-value) are provided.

### Association mapping

To find markers associated with the measured traits, both the GLM and the MLM models were used. The former evidenced associations between 170 markers and all analysed traits, except for *c*-LYC (Additional file 8). The mixed model, which takes account of the kinship matrix and genetic structure (K + Q), was preferred since familial relationships and population structure were found in the studied collection. In the MLM + Q + K method, the genetic structure with co-ancestry matrix Q = 3 was used, following STRUCTURE results. Table 3 summarizes the results of significant associations obtained by the TASSEL program after Bonferroni correction and using two different MAF thresholds (>5% and >10%). At MAF >5% the analysis revealed only one marker associated with pH, two markers with PHE, three with AsA, β-C and *t*-LYC, six with FW and TA. No marker was found associated with *c*-LYC, DMW and SSC. In order to confirm the associations with *loci* exhibiting strong allelic effects, results at MAF >10% were also provided. A total of 11 out of 24 markers were confirmed, and at least one marker still resulted significantly associated with each trait. In particular, markers associated with AsA, PHE and pH were all confirmed at both MAF thresholds, whereas the number was strongly reduced for traits, such as FW and TA.

**Table 3:** **Association statistics of markers significantly associated with seven traits by Mixed Linear Model (MLM) with two different MAF thresholds (5% and 10%)**

**ASSOCIATION STATISTICS**									
						**MAF >5%**		**MAF >10%**	
Trait^a^	Marker Index	SolCap ID	Gene^b^	Ch	Site bp	p value	R^2^	p value	R^2^
**AsA**	2383	solcap_snp_sl_20936	Solyc03g112630.2.1	3	57066578	2.74E-04	0.140	1.30E-04	0.145
	7588	solcap_snp_sl_9377	Solyc03g112670.2.1	3	57099944	2.74E-04	0.140	1.30E-04	0.145
	1241	solcap_snp_sl_105	Solyc05g052410.1.1	5	61782821	3.92E-04	0.179	4.35E-04	0.176
log **(β-C)**	2022	solcap_snp_sl_17063	Solyc01g087600.2.1	1	74314683	3.61E-04	0.198	4.58E-04	0.173
	2025	solcap_snp_sl_17072	Solyc01g087670.2.1	1	74360789	2.44E-04	0.206		
	2028	solcap_snp_sl_17076	Solyc01g087880.2.1	1	74515488	4.94E-04	0.192	2.48E-04	0.185
log **(t-LYC)**	3525	solcap_snp_sl_27094	Solyc03g031480.2.1	3	8291198	1.82E-04	0.175		
	3526	solcap_snp_sl_27099	Solyc03g031820.2.1	3	8571009	1.82E-04	0.175		
	3104	solcap_snp_sl_24679	ND	10	60360427	2.38E-04	0.203	9.66E-05	0.208
log **(PHE)**	354	solcap_snp_sl_100367	Solyc08g082350.2.1	8	62345755	6.2E-04	0.147	7.62E-05	0.213
	4365	solcap_snp_sl_34253	Solyc11g010170.1.1	11	3259108	5.13E-05	0.198	2.03E-04	0.150
log **(FW)**	2992	solcap_snp_sl_23884	Solyc02g078790.2.1	2	38009446	4.49E-04	0.175		
	2272	solcap_snp_sl_19779	Solyc08g006170.1.1	8	886583	2.04E-04	0.165		
	2273	solcap_snp_sl_19780	Solyc08g006170.1.1	8	886634	2.04E-04	0.165		
	2274	solcap_snp_sl_19782	Solyc08g006170.1.1	8	887192	2.04E-04	0.165		
	2275	solcap_snp_sl_19783	ND	8	887435	3.49E-06	0.239		
	1081	solcap_snp_sl_44897	Solyc11g071840.1.1	11	52280165	5.66E-04	0.132	1.57E-04	0.170
log **(pH)**	2246	solcap_snp_sl_19556	Solyc11g017070.1.1	11	7863387	2.65E-04	0.168	5.27E-04	0.131
log **(TA)**	955	solcap_snp_sl_54697	Solyc01g107550.2.1	1	86813075	4.59E-06	0.254		
	2032	solcap_snp_sl_17161	Solyc02g084520.2.1	2	42190707	5.25E-04	0.156	4.83E-04	0.137
	443	solcap_snp_sl_100446	Solyc03g083440.2.1	3	46891412	6.52E-04	0.149		
	3999	solcap_snp_sl_30911	Solyc03g093310.2.1	3	47931799	4.15E-04	0.159		
	1210	solcap_snp_sl_101075	ND	4	222976	1.98E-04	0.175		
	1010	solcap_snp_sl_45282	Solyc04g005510.2.1	4	344863	1.98E-04	0.175		

Marker index, SolCAP ID, corresponding gene, locus position (Ch and site), p-value and marker R^2^ are reported for each marker.

^a^ *AsA*: Ascorbic Acid, *β-C*: β-carotene, *t-LYC*:*trans*-lycopene, PHE:phenolics, *FW*: Fresh weight, *TA*: Titratable acidity. ^b^ *ND* = Not detected gene for the marker.

AsA content was associated with markers 2383 and 7588, which map on chromosome 3 spanning a region of 150 kbp, and with marker 1241 on chromosome 5. For markers on chromosome 3, genotypes with major alleles showed an increasing AsA level, compared to genotypes with minor alleles (Additional file 9). By contrast, for marker 1241 the minor allele incremented the phenotype. For β-carotene, the analysis revealed significant associations for markers 2022, 2025 and 2028 mapping on chromosome 1. Each markers explained approximately 20% of the phenotypic variation and the minor alleles in all cases contributed to enhance values. Markers 3525 and 3526, co-localized on chromosome 3, and marker 3104 mapping on chromosome 10, were associated with *t*-LYC with R^2^ values of 0.175 and 0.150, respectively. In all cases, the major alleles showed a very high effect with respect to the corresponding minor alleles. PHE was associated with markers 354 on chromosomes 8 and 4365 on chromosome 11. In both cases, the minor alleles increased the metabolite content.

FW was associated with six markers when the MLM was applied using MAF > 5%: the first was 2992 on chromosome 2, which explained about 17% of the phenotypic variation. Three markers co-segregated on chromosome 8, and mapped in the same gene (Solyc08g006170.1.1). The fifth marker 2275 explained the largest phenotypic variation (R^2^ = 0.239), and mapped 300 bp downstream to Solyc08g006170.1.1. The last marker was 1081 on chromosome 11, and it was the only confirmed using MAF >10%. Moreover, since the multiple regression analysis evidenced a great impact of the genetic structure only on FW, for this trait the association analysis was carried out also on the three separate Q sub-populations. Results confirmed that association of marker 1081 is maintained within the sub-populations (Q1, p-value = 1.90 E-05; Q2, p-value = 4.88E-05; Q3, p-value = 4.7E-02), suggesting that the association of this marker could be considered adequately robust. TA was associated with marker 955 on chromosome 1, which explained about 25% of the phenotypic variation (R^2^). The other five markers explaining the remaining part of phenotypic variation were marker 2032 mapping on chromosome 2, markers 443 and 3999 co-localized on chromosome 3, and markers 1010 and 1210 on chromosome 4. Finally, only one significant SNP was associated with pH, and explained 16.8% of phenotypic variation. Genotypes exhibiting minor allele for all markers associated with TA and pH had significantly higher value than genotypes with the major allele.

**Figure 2:**
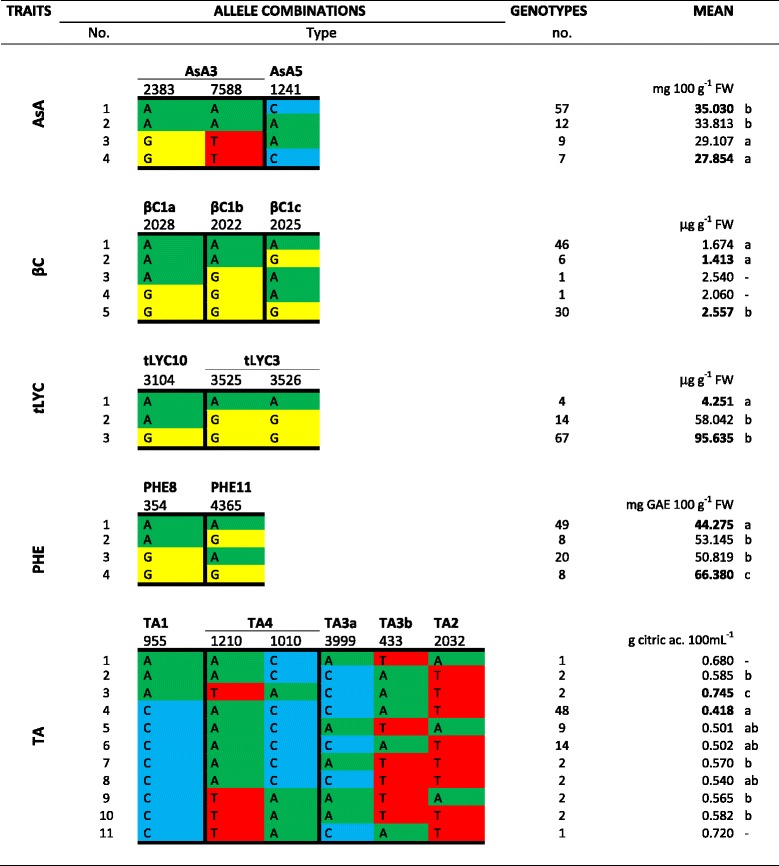
**Allele combinations at markers associated with each trait.** Number and type of allele combinations, number of genotypes and their mean phenotypic values are shown. Significant differences between groups were assayed by Duncan’s test. AsA = Ascorbic Acid, βC = β-carotene, tLYC = trans-lycopene, PHE = phenolics, TA = titratable acidity.

Finally, we evaluated the effect of different allele combinations at *loci* that were significantly associated with each trait (Figure 2). For each trait, mean and statistical significance among the groups of genotypes were calculated for all the allelic combinations. For AsA, four allele combinations were found. Group 1 showed the highest value (35.03 mg 100 g^−1^ FW, average of 57 genotypes) and group 4 the lowest (27.85 mg 100 g^−1^ FW, average of seven genotypes). For β-C, 46 genotypes in group 1 and six in group 2 had allele combinations associated with a low content and 30 genotypes with a high content. *t*-LYC showed three allele combinations. Four genotypes with yellow fruits belong to group 1 associated with the lowest lycopene content (4.25 μg g^−1^ FW mean value), whereas 67 genotypes showed an allele combination associated with high lycopene content (95.63 μg g^−1^ FW mean value). For PHE, four groups were observed. The largest was group 1, including 49 genotypes and showing the minimum amount of phenolics (44.27 mg GAE 100 g^−1^ FW mean value), while the group associated to the maximum amount (66.38 mg GAE 100 g^−1^ FW mean value) included eight genotypes. Eleven allele combinations were identified for titratable acidity and the one associated with the highest value (0.745 g citric ac. 100 mL^−1^ of juice) was detected in two genotypes, whereas that associated with the lowest value (0.418 g citric ac. 100 mL^−1^ of juice) was detected for a group of 48 genotypes. Intermediate values were detected for the other nine groups.

## Discussion

Results of association mapping studies depend on different factors, including type and size of mapping population, trait investigated, number of environments and years used for phenotyping, and type and genome coverage of molecular markers. The present study took into account a collection of cultivated tomato genotypes, including mainly Italian landraces but also Latin American and other worldwide-spread landraces and varieties. Genotypes were selected for the high variability of fruit morphological traits, such as size, shape, skin and flesh colour (data not shown), whereas little or no information was available regarding their nutritional and quality traits. Population structure and familial relationships, likely due to local adaptation, selection and breeding history, were found in the collection. Large populations are desirable for association mapping studies in order to obtain a high power to detect genetic effects of moderate size [10,23]; however, there is a high cost associated with genotyping and phenotyping such populations, particularly for traits requiring extensive field trials, chemical or biochemical assays and a number of replications for measures’ reliability. Therefore, we assumed that the size of our tomato collection was adequate for association mapping studies, as previously reported for bean [24], peanut [25] and barley [26], as well as for tomato [14], in analyses that involved approximately 90 genotypes.

**Figure 3:**
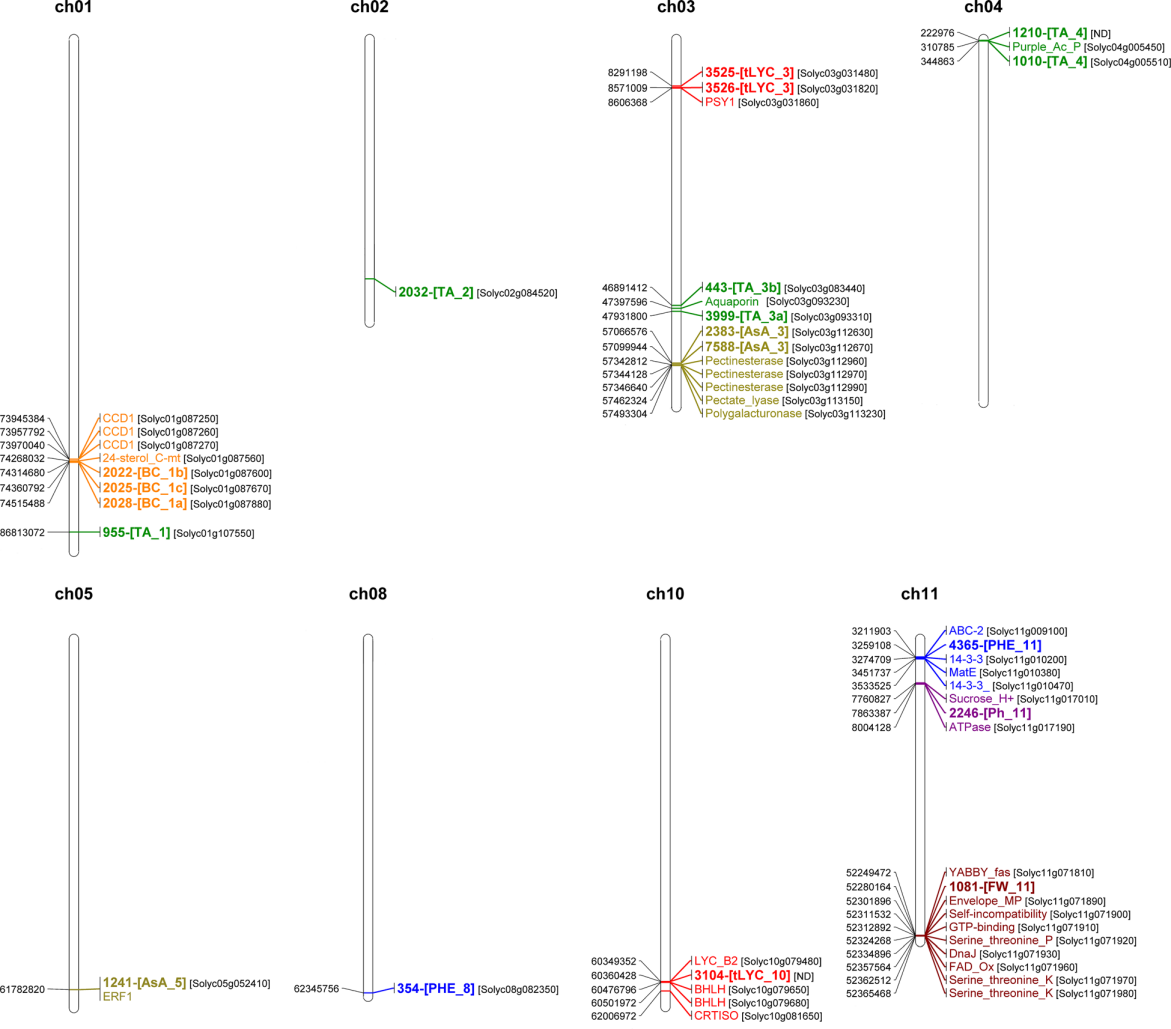
**Map of 24 markers significantly associated with seven phenotypic traits and of co-localized candidate genes for trait variation.** Position in bp for each marker/gene is shown at the left side of each chromosome. Each colour represents a trait. Significantly associated markers and the corresponding trait are shown in bold. BC = β-Carotene; FW = Fresh Weight; tLYC = *trans*-Lycopene; TA = Titratable Acidity; AsA = Ascorbic Acid; PHE = Phenolics; 24-sterol_C_mt = 24-sterol C-methyltransferase; CCD1 = Carotenoid cleavage dioxygenase 1; PSY1 = Phytoene synthase 1; Purple_Ac_P = Purple acid phosphatase; ERF1 = Ethylene responsive factor 1; TF-B3a = transcriptional factor B3a; LYC_B2 = Lycopene Beta cyclase; CRTISO = Prolycopene isomerase; FAD_Ox = FAD-linked sulfhydryl oxidase.

**Figure 4:**
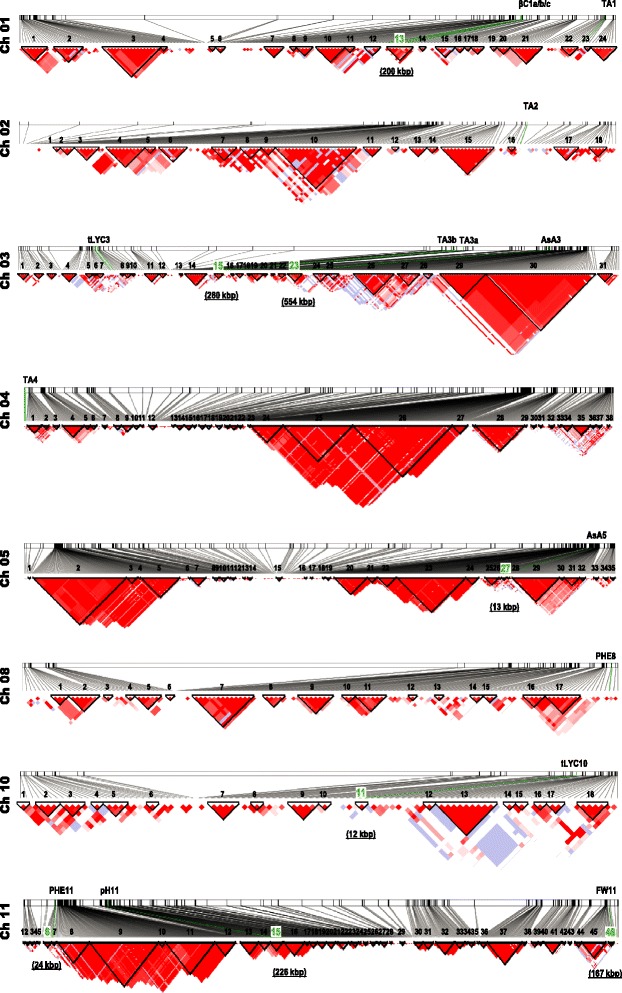
**LD Blocks for chromosomes where associated markers were localized.** Blocks of markers that are in strong LD using confidence intervals algorithm in Haploview software (black triangle) are reported. The size of blocks (in kbp) in which significantly associated markers fall (green lines) is shown. The colour scheme (D’/LOD) used to represent pairwise LD estimate ranges from bright red (LOD ≥2 and D’ = 1) to white ( LOD <2 and D’ <1).

Using the MLM and the MAF threshold >5%, 24 SNPs associated with seven out of ten traits were identified, even though the GLM detected a higher number (170 SNPs) of markers associated with nine traits. Since previous works highlighted the greater efficiency of the K + Q model in correcting spurious associations in tomato populations [14] and other species [11,27,28], in order to reduce the amount of false-positives our focus was on the highly significant associations detected by the MLM. Among the 24 SNPs, four associated with FW were then excluded from subsequent analyses, since they were highly influenced by the population structure. In addition, in order to obtain a powerful confirmation of the 20 SNPs associated in the present study, our analysis included results obtained with the MAF threshold higher than 10%, following the strategy reported in recent studies carried out in tomato, where this threshold was preferred [15,20]. As a result of this second analysis, 11 SNPs were confirmed. However, since MAF >5% is the most widely used in association mapping studies and in our opinion it constitutes a good compromise between the reduction of false positives and the loss of rare alleles, we will discuss the phenotypic variation for the traits analysed in terms of the potential involvement of all 20 SNPs significantly associated in our study. A detailed map of markers and putative genes responsible for each trait variation is presented in Figure 3, and LD blocks onto which significant associations fall, obtained by HAPLOVIEW software, are shown in Figure 4.

### Nutritional traits

Concerning antioxidants traits, markers associated with AsA, β-C, *t*-LYC and PHE were searched for, since these are bioactive compounds exhibiting beneficial effects on human health [29]. In particular, three markers (2383, 7588 and 1241) associated with AsA were identified, which differed from those detected by Sauvage et al. [20] using a similar GWAS approach, but exploiting accessions belonging to different tomato species. Two markers we identified corresponded to genes Solyc03g112630.2.1 and Solyc03g112670.2.1 mapping on chromosome 3 and were annotated as *Fas-associated factor 1-like* and Genomic DNA chromosome 5 P1, respectively. The other gene (Solyc05g052410.1.1) was located on chromosome 5 and annotated as *Ethylene-responsive transcription factor 1* (*ERF1*). The Fas-associated factor 1-like protein is involved in an apoplastic mechanism and no direct evidence was reported to correlate its function with AsA accumulation. Since no specific functions were also assigned to Solyc03g112670.2.1, it was thought that the polymorphisms identified in this region of chromosome 3 could be in LD with other candidate genes. In order to verify this hypothesis, a scan was performed of the surrounding genomic area in LD with markers 2383 and 7588. A cluster of pectinesterases (120 kbp from marker 7588), one pectate lyase (240 kbp from marker 7588) and one polygalacturonase (350 kbp from marker 7588) were detected in LD block 23 on chromosome 3. These findings suggest that the alternative D-galacturonic biosynthetic pathway could contributes to regulate AsA variation in the tomato population under study, as previously reported in tomato [30] and other species [31,32]. In addition, concerning the *ERF1* gene, Di Matteo and colleagues [30] showed that in one *S. pennellii* introgression line a different expression of genes associated with ethylene biosynthesis might trigger pectin degradation resulting in AsA accumulation. Taken together, these results suggest a possible regulation of genes associated with markers 2383 and 7588 (related to pectin degradation) *via* Ethylene Responsive Factor 1 associated with marker 1241.

*Cis* and *trans* isomers of lycopene derive from a cascade of enzymatic reactions taking place in plastids [33]. Intermediates in the first part of the pathway are *cis*-configured. A pro-lycopene isomerase (CrtISO) then produces all-*trans*-lycopenes from tetra-*cis*-lycopene. Subsequent reactions convert *trans*-lycopene into β-carotene by the action of a lycopene β-cyclase (β-Lcy). Although no associations were detected for *c-*LYC, three significant associations with *t*-LYC were identified. Markers 3525 and 3526, co-localized on chromosome 3, matched a *putative metallocarboxypeptidase inhibitor* (Solyc03g031480.2.1) and *tyrosyl-DNA phosphodiesterase* (Solyc03g031820.2.1) whereas marker 3104 on chromosome 10 did not match annotated genes. Interestingly, even if they are not directly linked to the *trans*-lycopene content, analysis of the genomic area highlighted the presence of a *phytoene synthase 1* (Solyc03g031860.2.1) close to markers 3525 and 3526 (at 315 and 24 kbp, respectively) and a *lycopene β-cyclase 2* (Solyc10g079480.1.1) at 9 kbp from marker 3104. This showed that the association mapping approach used was able to validate two candidate genes already known to be involved in the carotenoid pathway. In fact, the identified *phytoene synthase 1*, which catalyzes a rate-limiting step in the carotenoids pathway, corresponds to the locus “*r*” [34] that carries a recessive mutation conferring a characteristic yellow flesh phenotype. Four accessions in the population showed a genotype associated to the locus “*r*” and all have yellow flesh fruit as a consequence of a low *trans*-lycopene content. In addition, on chromosome 10, besides the lycopene β-cyclase, also a carotene isomerase (CrtISO, Solyc10g081650.1.1), which converts pro-lycopene to *trans*-lycopene [35], was localized 1.2 Mbp downstream marker 3104 (Figure 3). As concerns β-C, significant associations were found on chromosome 1 with Solyc01g087600.2.1 annotated as Protein E03H4.4, Solyc01g087670.2.1 annotated as a guanine nucleotide-binding protein, involved in blue light perception signal pathways [36], and Solyc01g087880.2.1, which has no homology with any gene of known function. These results prompted to investigate alternative genes in this region. Scanning the genomic area associated to these three markers (LD block 13 on chromosome 1), a *24-C-sterol-methyltransferase* was found that is involved in steroid biosynthesis. Moreover, a cluster of three *carotenoid cleavage dioxygenase 1* (*CCD1*) genes was also identified at 300 kbp from marker 2022. *CCD1* genes cleave the carotenoid substrate at different double bonds to produce terpenoid flavour volatiles (apocarotenoids) that contribute to the overall aroma and taste of tomato fruit [37]. It is hypothesized that the variation in the carotenoid pool may depend on the metabolic flux towards the cleavage reactions to produce apocarotenoids, but further functional experiments that will validate this hypothesis are required.

Finally, two polymorphic markers significantly associated with PHE were identified: marker 354 (Solyc08g082350.2.1), which encodes for a protein of unknown function, and marker 4365 (Solyc11g010170.1.1), encoding for a LanC-like protein2, which is involved in the modification and transport of peptides in bacteria [38]. However, no well-defined functions were reported for the latter gene in plants [39], even if a probable involvement as one receptor for abscisic acid (ABA) was hypothesized [40]. No gene of the phenolics pathways was detected in the putative region in association with marker 354. By contrast, significant co-localizations (LD block 6) found with marker 4365 included transport genes encoding two 14-3-3 proteins (Solyc11g010200 and Solyc11g010470), an ABC-2 transporter (Solyc11g009100) and a MATE efflux family protein (Solyc11g010380). The involvement of these transporters in enhancing the vacuolar compartmentalization of phenolic compounds was previously reported by Gomez *et al*. [41] and Di Matteo *et al*. [42], suggesting their probable role in the metabolism of this trait. A previous work [43] also identified QTLs for phenolic content in regions of chromosome 8 and 11 close to the markers detected, confirming the involvement of these regions in phenolics control.

### Quality traits

Major fruit quality traits of interest for both the fresh market and processing tomatoes include fruit size, shape, total solids, colour, firmness, ripening, pH, titratable acidity, soluble solids content and dry matter. In this study, a large number of associations with FW and TA were found, only one association with pH and no association with SSC and DWM.

FW is a quantitatively inherited trait controlled by up to 28 QTLs, even though QTL analyses in previous studies revealed that most (67%) phenotypic variation in fruit size could be attributed to six major loci (*fw1.1*, *fw1.2*, *fw2.1*, *fw2.2*, *fw3.2* and *fw11.3*) localized on chromosomes 1, 2, 3 and 11 [44-47]. The present study confirmed only one of the above loci (*fw11.3).*

Indeed, on chromosome 11 marker 1081 matched Solyc11g071840.1.1, annotated as a calmodulin binding protein, and was located in the LD block 40 that spans an interval of 167 kbp. This region contains both a portion of the *fw11.3* locus (starting 20 kbp downstream of marker 1081) and the *fas-YABBY* locus (24 kbp upstream of the marker), previously hypothesized to determine fruit size [48]. Therefore, the findings here reported not only confirmed the involvement of locus *fw11.3* in FW variation but also restricted the region of putative candidate genes with respect to the previously identified region of 149 kbp, which included 22 predicted genes [48]. Indeed, considering the LD block strategy used, marker 1081 was strongly associated only with a portion of *fw11.3* of around 70 kbp (52,301,894 – 52,365,467), including eight predicted genes. This region must be enriched in SNPs to locate precisely one or more responsible polymorphisms associated to the trait and further investigation should be carried out to fine map the putative gene responsible for the trait variation.

Among important quality traits in tomato, TA influences shelf-life of processed tomato and low pH values reduce the risk of pathogen growth in tomato products [49]. One locus associated with pH and five loci associated with TA were evidenced. Only marker 2246 that matches Solyc11g017070.1.1 on chromosome 11 (encoding for an eukaryotic translation initiation factor 3 subunit 2) proved associated with pH trait. An *ATPase* and a *Sucrose_H+ symporter* co-localized with this marker; they are proton pumps responsible for acidifying cellular compartment and might correlate with this trait. Moreover, previous studies identified a pH QTL in this region [44,50] supporting our hypothesis and confirming the possible involvement of this region in regulating the pH level in tomato fruit.

The most significant association with TA was with marker 955, which explains around 25% of the variation. The marker matched the predicted gene Solyc01g107550.2.1, which encodes for a methylthioribose kinase, an enzyme involved in recycling of methionine through the methylthioadenosine (MTA) cycle. The marker did not fall in any significant LD block on chromosome 1 and for this reason a narrow area around the marker was investigated to look for candidate genes. Four annotated genes not related to the trait under study and various unknown genes were identified, leaving the mechanism responsible for TA variation of this locus still obscure. Other significant associations were found on chromosome 3 for markers 443 and 3999 matching Solyc03g083440.2.1 (Glutamate synthase) and Solyc03g093310.2.1 (F-box family protein), respectively, in a region where previous studies detected QTLs for TA [44,50,51]. Markers 443 and 3999 are 1 Mbp away from one another and do not fall in the same LD block. Marker 443 falls in the 280 kbp LD block 15 that includes at least 40 genes, none of which might be so far involved in TA determination. On the other hand, for marker 3999 no significant LD block was inferred and few putative co-localizations could be highlighted. In particular, an *aquaporin* (Solyc03g093230.2.1) was identified as a potential candidate. The involvement of the aquaporin gene family in the modulation of fruit acidity was hypothesized in tomato antisense experiments, indicating a strong effect of this protein on the sugar/organic acid ratio in fruit [52]. An additional locus for TA was found on chromosome 4 between markers 1210 and 1010. This region includes about 30 genes and we pointed out a *purple acid phosphatase* (Solyc04g005450), a metalloenzyme that hydrolyses phosphate esters and anhydrides under acidic conditions [53]. No relevant co-localizations were instead found for marker 2032 on chromosome 2.

## Conclusions

The association mapping approach undertaken allowed detection of 20 SNPs associated with seven traits that are essential for breeding work aimed at improving nutritional and quality traits in both fresh market and processing tomatoes. The findings suggest that the use of a high marker density array and a highly efficient statistical model (K + Q) were suitable for detecting associations with the traits considered. Indeed, the co-localization of a group of associated loci with formerly identified candidate genes/QTLs validated the approach chosen, as evidenced for markers related to *t*-LYC, β-C content and FW. Consequently, it can be argued that all the SNPs identified might be exploited in the future as markers targeting the specific desirable phenotype for assisted selection. This is noteworthy if considering the different allelic combinations we identified for some traits, whose pyramiding would further enhance tomato fruit quality of the improved genotypes. A further validation in independent accessions panels or bi-parental populations would be in any case desirable.

In addition, a number of new putative candidate genes were detected in the genomic area in linkage disequilibrium with markers that were not functionally congruent with the trait to which they were associated with. These promising genes might be involved in the pathways controlling the biosynthesis and accumulation of the metabolite/trait analysed, as in the case of a group of genes related to cell wall metabolism that might be hypothesized to contribute to a higher AsA content in tomato fruit or a group of vacuolar transporters that might regulate the accumulation of phenolic compounds. In order to detect the functional SNPs determining the different phenotypes, the identified candidate genes are being investigated by a target resequencing approach in a group of varieties belonging to the same collection. Finally the role of these new candidate genes will be validated in the future by functional genomics approaches.

## Methods

### Plant material

Plant material consisted of 96 tomato genotypes, including Italian and Latin American landraces, and vintage and modern varieties collected from seed banks in Italy and worldwide. In detail, accessions were derived from Italian breeders’ collections, Regional and National Italian Institutions (Regione Campania, ARCA2010 Cooperative for Agriculture, MiPAF-CRA Centre for Research in Agriculture, University of Naples Federico II), and International Institutions (Plant Genetic Resources Unit, USDA, Tomato Genetics Resource Center, Davis, USA, Hebrew University of Jerusalem, Israel). All genotypes were grown during the seasons 2011 and 2012, according to a randomized complete block design with three replicates (10 plants *per* replicate), in field plots at the Agricultural Experiment Station of CRA-ORT in Battipaglia (Salerno, Italy). Field trials were conducted in accordance with the Italian legislation. All genotypes were subject to phenotypic and genotypic analyses. Out of 96 genotypes, six were excluded from phenotyping, since they produced few fruits or segregated for some morphological traits. Five additional genotypes were filtered out since were considered unreliable due to the large number of low quality SNP scores. Therefore, the number of samples finally used for association mapping was reduced to 85.

### Phenotyping

Chemical and physical traits were evaluated on ten fruits harvested at red ripe stage for each biological replicate as recommended in the SCAR Agro-Food Tomato Working Group [54]. Traits included fresh weight (FW), total dry matter weight (DMW), soluble solids content (SSC), titratable acidity (TA) and pH.

Metabolic analyses were carried out on collected fruits stored at −80°C (three replicates of 5–8 fruits per accession): ascorbic acid (AsA), β-carotene, *trans*- and *cis*-lycopene, and phenolics content were measured as described below. AsA content was determined as reported by Di Matteo *et al*. [30] with minor modifications. Briefly, 500 mg of frozen powder were added to 300 μL of ice-cold 6% trichloroacetic acid (TCA) in 2 mL Eppendorf tubes. Samples were vortexed and left on ice for 15 min, and then centrifuged for 15 min at 25,000 g at 4°C. The supernatant was transferred to a clean tube and 20 μL were used for the assay, as described in the manuscript cited above. The AsA content was expressed as mg *per* 100 g^−1^ of FW.

Extraction and analysis of carotenoids were carried out on 5 g of fruit pericarp, according to Ishida *et al*. [55]. Reversed Phase-High Performance Liquid Chromatography (RP-HPLC) analysis was performed through a Waters E-Alliance HPLC system constituted by a 2695 separations module with quaternary pump, autosampler, and a 2996 photodiode array detector; data were acquired and analyzed with Waters Empower software. The chromatographic separations were performed at a flow rate of 0.8 mL min^−1^ and at 0.005 AUFS (Absorbance Units Full Scale) by using a reversed phase, analytical polymeric C30 column (250 × 4.6 mm i.d.; 3 μm particle diameter; YMC, Wilmington, NC, USA). Results were expressed as μg g^−1^ of FW. Solvents used for sample preparation and extractions were of analytical grade, while those for HPLC analysis (methyl-t-butyl ether, methanol, ethyl acetate and tetrahydrofuran) were of HPLC grade; all were obtained from Merck (Darmstadt, Germany). *Trans*-carotenoid standards (lycopene and β-carotene) used in HPLC analyses were purchased from Sigma Chemical Co (Sigma-Aldrich Company, St. Louis, MO, USA).

Total phenolics content was assayed using a modified procedure of the Folin–Ciocalteu test [56]. In brief, 250 mg of frozen ground tissue were homogenized in a mortar with pestle and extracted using 1 mL of 60% methanol. Samples were left on ice for 3 min in the dark. Crude extracts were transferred into a 15 mL tube and volume was increased to 5 mL by adding 60% methanol. The samples were centrifuged at 3000 *g* for 5 min; afterwards, 62.5 μL of the supernatant, 62.5 μL of Folin–Ciocalteu’s reagent (Sigma) and 250 μl of deionized water were mixed and incubated for 6 min; 625 μL of 7.5% sodium carbonate and 500 μL of deionized water were added to the samples and incubated for 90 min at room temperature in the dark. Absorbance was measured at 760 nm. The concentration of total phenolics was expressed in terms of mg of gallic acid equivalents (GAE) *per* 100 g^−1^ of FW.

For each year, the normal distribution of data was verified using a Shapiro-Wilk test. Six (β-C, PHE, FW, pH, SSC, TA) out of 10 traits were not normally distributed and were log_10_ transformed before performing the association mapping analysis. In order to test the consistency of phenotypic characterization over years, heritability values were calculated as reported in [15]. Moreover, year and genotype effects were assessed by two-factor analysis of variance. Since the genetic effect over the two years was more significant than the genotype x year interaction for all traits, associations were calculated by using means over years. Pearson correlation coefficients were calculated among all pairs of traits. Analyses were carried out with the R program [57].

### SNP genotyping

Samples were genotyped using a tomato array built in the framework of the Solanaceae Coordinated Agricultural Project (SolCAP) from NIFA/USDA and based on the ILLUMINA Infinium Technology. The SolCAP tomato panel includes 7,720 markers constructed on eSNPs deriving from six tomato genome sequences. Details of the SolCAP SNP discovery pipeline are described in Hamilton *et al*. [2] and Sim *et al*. [6]. Information about SolCAP SNPs are available in the SGN database (http://solgenomics.net/).

For each accession, genomic DNA was extracted from fresh, young leaf tissue using the DNeasy Plant Mini kit (QIAGEN, Valencia, CA) according to the manufacturer’s recommendations. DNA quality and concentration were evaluated on agarose gel and spectrophotometrically using the Nanodrop instrument (Thermo Fisher Scientific, Wilmington, USA). Genotyping was conducted at the Genomix4Life S.r.l (http://www.genomix4life.com) using 250 ng of DNA per accession following the manufacturer’s protocol for the Illumina Infinium assay. Intensity data and SNP calls were performed by GenomeStudio version 1.7.4 (Illumina Inc., San Diego, CA, USA). SNPs were called using the Infinium chip cluster file based on the SolCAP tomato collection and a manual classification was implemented when the default clustering was not clearly defined. In addition, quality and reproducibility were tested using duplicated DNA samples.

### Data analysis

The set of SNPs was filtered in order to perform molecular analyses. Markers with more than 10% missing genotypes and with minor allele frequency (MAF) <5% were removed. The physical position of all SNPs on the 12 tomato chromosomes was obtained from the SGN database (http://solgenomics.net).

Linkage disequilibrium (LD) values were calculated on SNP set with MAF greater than 5%. Pairwise *r*^2^ between markers was calculated for each chromosome using TASSEL v.4.0 [58]. Values of *r*^2^ were plotted against physical distance and LD decay was inferred *via* locally weighted scatterplot smoothing (LOWESS) using R software and testing smoothing parameters fixed to 0.2. In order to determine the distance of LD decay, for each chromosome different *r*^*2*^ baseline values (0.2, 0.3 and 0.5) were tested [59]. A Bayesian population classification was carried out using STRUCTURE 2.3.3 [60]. Since population structure estimates assume unlinked markers, a sub-set of 600 markers with an average distance of 600 kbp was chosen to perform STRUCTURE analysis. STRUCTURE runs were carried out with a length of burn-in and MCMC (Markov chain Monte Carlo) of 100,000 each. Twelve independent runs were conducted allowing K (number of populations) varying from 2 to 13. Optimal K was inferred by using the Evanno *et al*. [61] transformation method. The influence of the population structure on the phenotypic variation of considered traits was assessed by multiple regression analysis. Associations between genotypes and phenotypes were determined using the General Linear Model (GLM) and the Mixed Linear Model (MLM) in TASSEL v4.0. For both models the structured association model (Q model) was performed [62] and two different thresholds of MAF (>5% and >10%) were used. In the association analysis, we considered the kinship matrix based on the SNP data in the model of MLM, and the population structure covariates detected in the tomato accessions with the STRUCTURE analysis. The significance of association between traits and markers was estimated by using an adjusted *P* value (Bonferroni correction) and the threshold for the association was set to <5.88×10^−4^ (0.05/85). In addition, in order to estimate LD blocks according to the definition of Gabriel *et al*. [63], the HAPLOVIEW software [64] was used with the following parameters: MAF >0.05; Hardy–Weinberg P-value cut-off, 0; percentage of genotyped lines >0.75.

### Data availability

The list of genotypes including details on source and distribution, and the SNP genotype dataset are deposited on the LabArchives repository hosted http://dx.doi.org/10.6070/H4TT4NXN.

## Additional files

Additional file 1:
**Phenotypic values of the ten nutritional and quality traits analysed for each genotype.** Mean and standard error are reported. Additional file 2:
**Pearson correlation coefficients between the ten nutritional and quality traits analysed.**
Additional file 3:
**Distribution of SolCAP markers on 12 tomato chromosomes.** In red markers showing MAF < 5%, in blue markers showing MAF ≥ 5% (MAF: Minor Frequence Allele). Additional file 4:
**LD decay values at different fixed baselines (0.2, 0.3 and 0.5) for each chromosome.** Distance of LD decay was expressed in kbp. Additional file 5:
**LD decay inferred using locally weighted scatterplot smoothing (LOWESS) algorithm.** Values of r^2^ were plotted against physical distance. A fixed baseline of 0.2 was considered in order to determine distance of LD decay. Additional file 6:
**A) Population STRUCTURE based on a sub-set of 600 markers and B) optimal K inferred using the Evanno method.** Each vertical bar represents one of 85 individuals analysed. Bar length is proportional to the inferred ancestry values into each group for each individual. Additional file 7:
**Kinship matrix calculated at MAF >5% and MAF >10%.**
Additional file 8:
**Markers associated with the ten nutritional and quality traits using the General Linear Model (GLM).** Markers associated also by the Mixed Linear Model (MLM) are reported in bold. Additional file 9:
**Allele effect of markers significantly associated with seven traits by Mixed Linear Model (MLM) using a MAF threshold >5%.** For each marker the number of genotypes observed (Obs) and phenotypic effects (Effect) for both major and minor alleles are shown.
