# Supplementary material for: An association mapping approach to identify favourable alleles for tomato fruit quality breeding
Source: BMC Plant Biol. 2014 Dec 3;14:337. doi: 10.1186/s12870-014-0337-9 (PMC4266912; doi:10.1186/s12870-014-0337-9)
Supplement: Additional file 2: — Pearson correlation coefficients between the ten nutritional and quality traits analysed. [file 12870_2014_337_MOESM2_ESM.docx]

| **Trait** |  | AsA | β-C | t-LYC | c-LYC | PHE | DMW | FW | pH | SSC |
| --- | --- | --- | --- | --- | --- | --- | --- | --- | --- | --- |
| β-C |  | 0.08 | - |  |  |  |  |  |  |  |
| t-LYC |  | -0.10 | 0.07 | - |  |  |  |  |  |  |
| c-LYC |  | -0.15 | 0.07 | 0.89** | - |  |  |  |  |  |
| PHE |  | 0.55** | -0.09 | -0.38** | -0.49** | - |  |  |  |  |
| DMW |  | 0.12 | -0.06 | -0.10 | -0.15 | 0.06 | - |  |  |  |
| FW |  | -0.28** | -0.01 | 0.23* | 0.36** | -0.39** | -0.05 | - |  |  |
| pH |  | -0.13 | 0.03 | 0.18 | 0.12 | -0.23* | 0.13 | 0.10 | - |  |
| SSC |  | 0.47** | 0.06 | -0.15 | -0.20 | 0.42** | 0.07 | -0.35** | -0.04 | - |
| TA |  | 0.46** | 0.02 | -0.19 | -0.16 | 0.48** | -0.11 | -0.28** | -0.70** | 0.46** |

Additional file 2. Pearson correlation coefficients between the ten nutritional and quality traits analysed.

*, ** *P* < 0.05 and < 0.01, respectively

AsA: Ascorbic Acid, β-C: β-carotene, t-LYC:*trans*-lycopene, c-LYC: *cis*-lycopene, PHE: phenolics, DMW: dry matter weight, FW: fresh weight, SSC: soluble solids content, TA: titratable acidity
