# Supplementary figures and images for: An association mapping approach to identify favourable alleles for tomato fruit quality breeding
Source: BMC Plant Biol. 2014 Dec 3;14:337. doi: 10.1186/s12870-014-0337-9 (PMC4266912; doi:10.1186/s12870-014-0337-9)

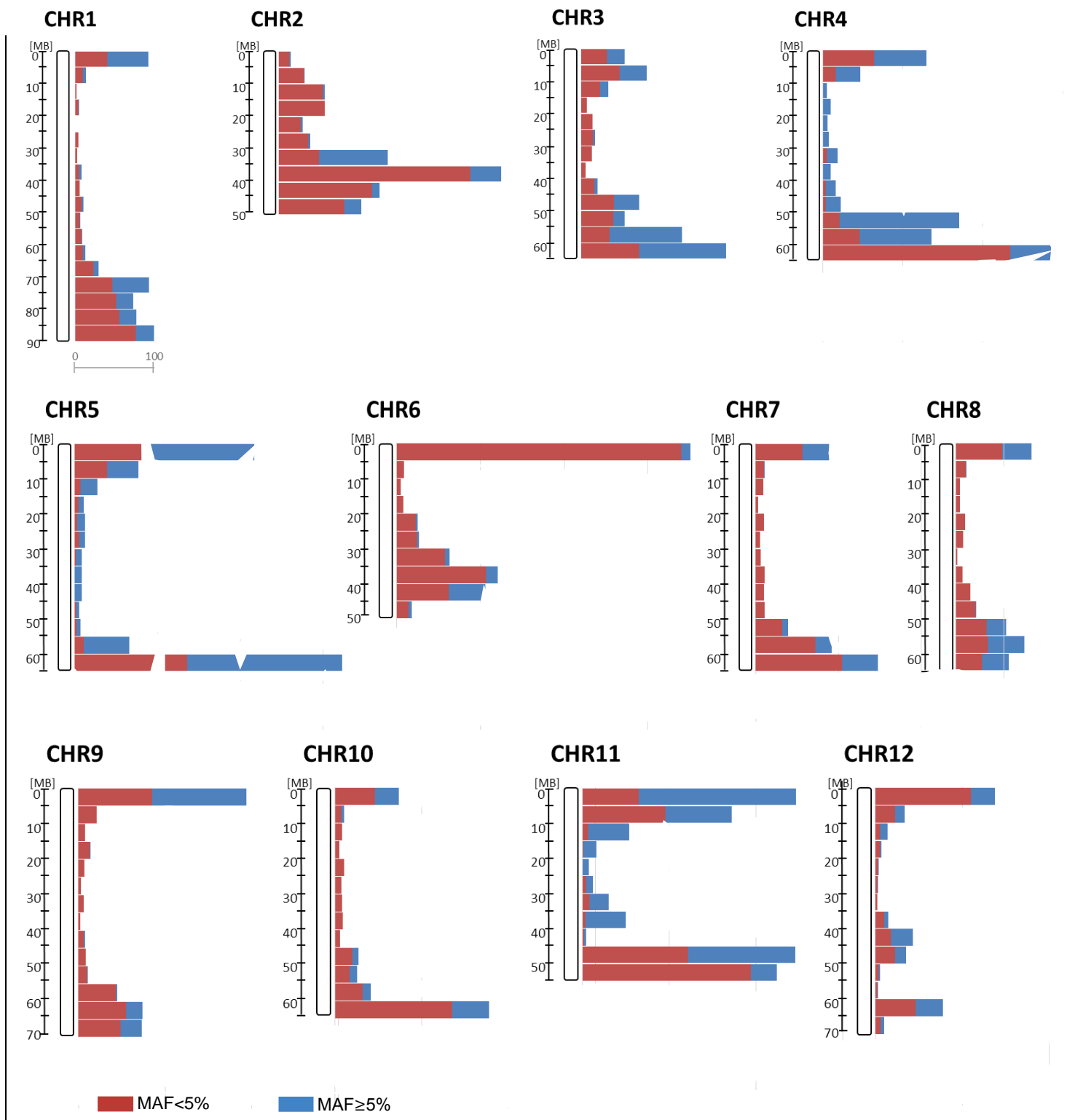

Supplement: Additional file 3: — Distribution of SolCAP markers on 12 tomato chromosomes. In red markers showing MAF < 5%, in blue markers showing MAF ≥ 5% (MAF: Minor Frequence Allele). [file 12870_2014_337_MOESM3_ESM.pdf]

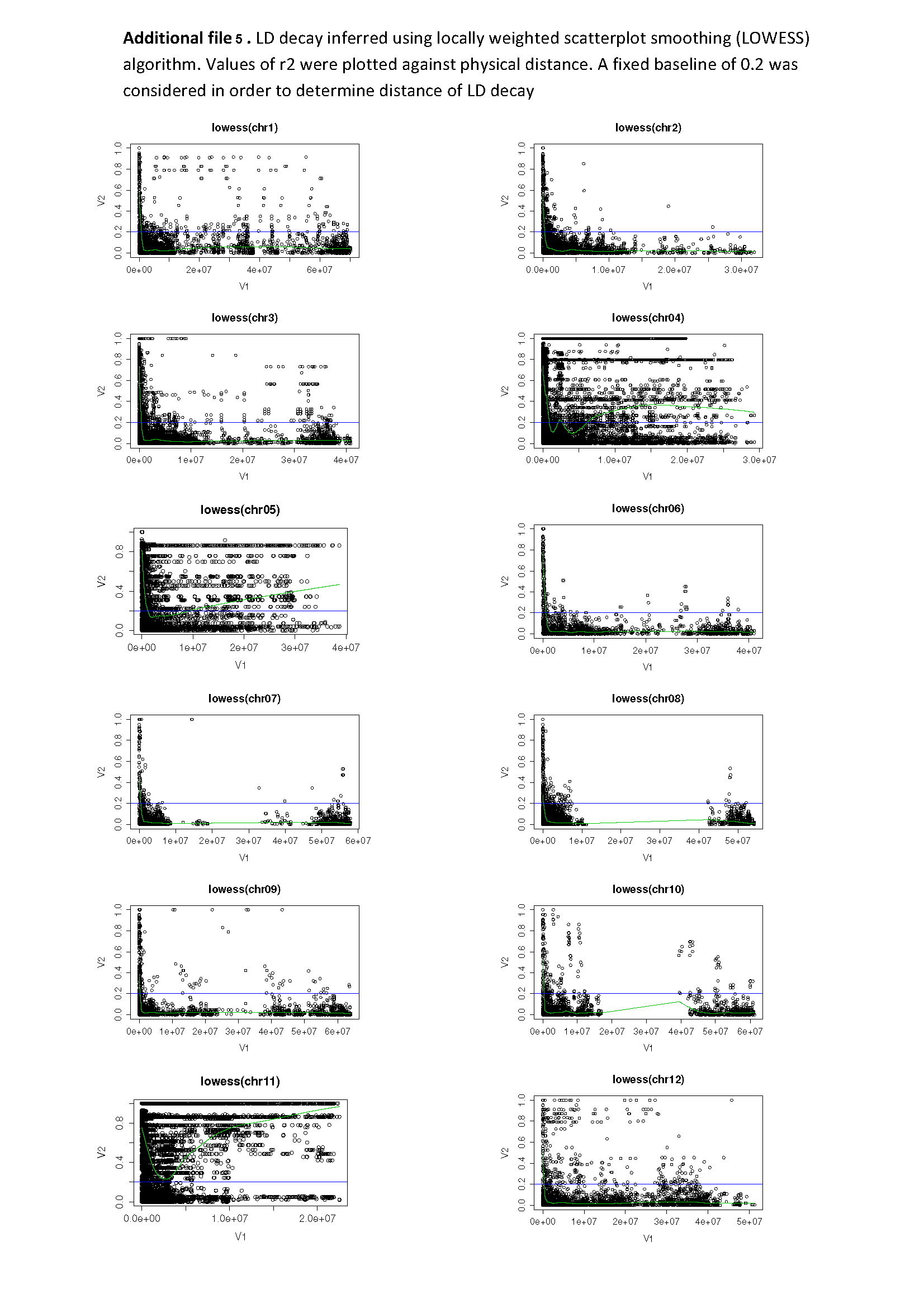

Supplement: Additional file 5: — LD decay inferred using locally weighted scatterplot smoothing (LOWESS) algorithm. Values of r2 were plotted against physical distance. A fixed baseline of 0.2 was considered in order to determine distance of LD decay. [file 12870_2014_337_MOESM5_ESM.tiff]

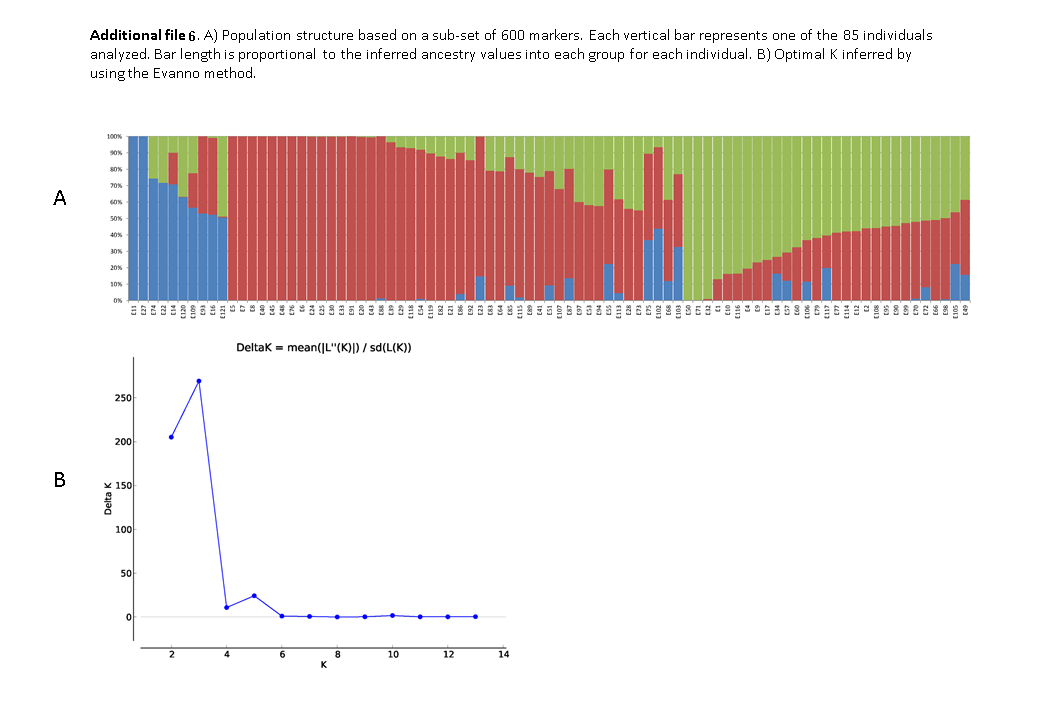

Supplement: Additional file 6: — A) Population STRUCTURE based on a sub-set of 600 markers and B) optimal K inferred using the Evanno method. Each vertical bar represents one of 85 individuals analysed. Bar length is proportional to the inferred ancestry values into each group for each individual. [file 12870_2014_337_MOESM6_ESM.png]
