# Supplementary material for: An association mapping approach to identify favourable alleles for tomato fruit quality breeding
Source: BMC Plant Biol. 2014 Dec 3;14:337. doi: 10.1186/s12870-014-0337-9 (PMC4266912; doi:10.1186/s12870-014-0337-9)
Supplement: Additional file 4: — LD decay values at different fixed baselines (0.2, 0.3 and 0.5) for each chromosome. Distance of LD decay was expressed in kbp. [file 12870_2014_337_MOESM4_ESM.ppt]

## Slide 1
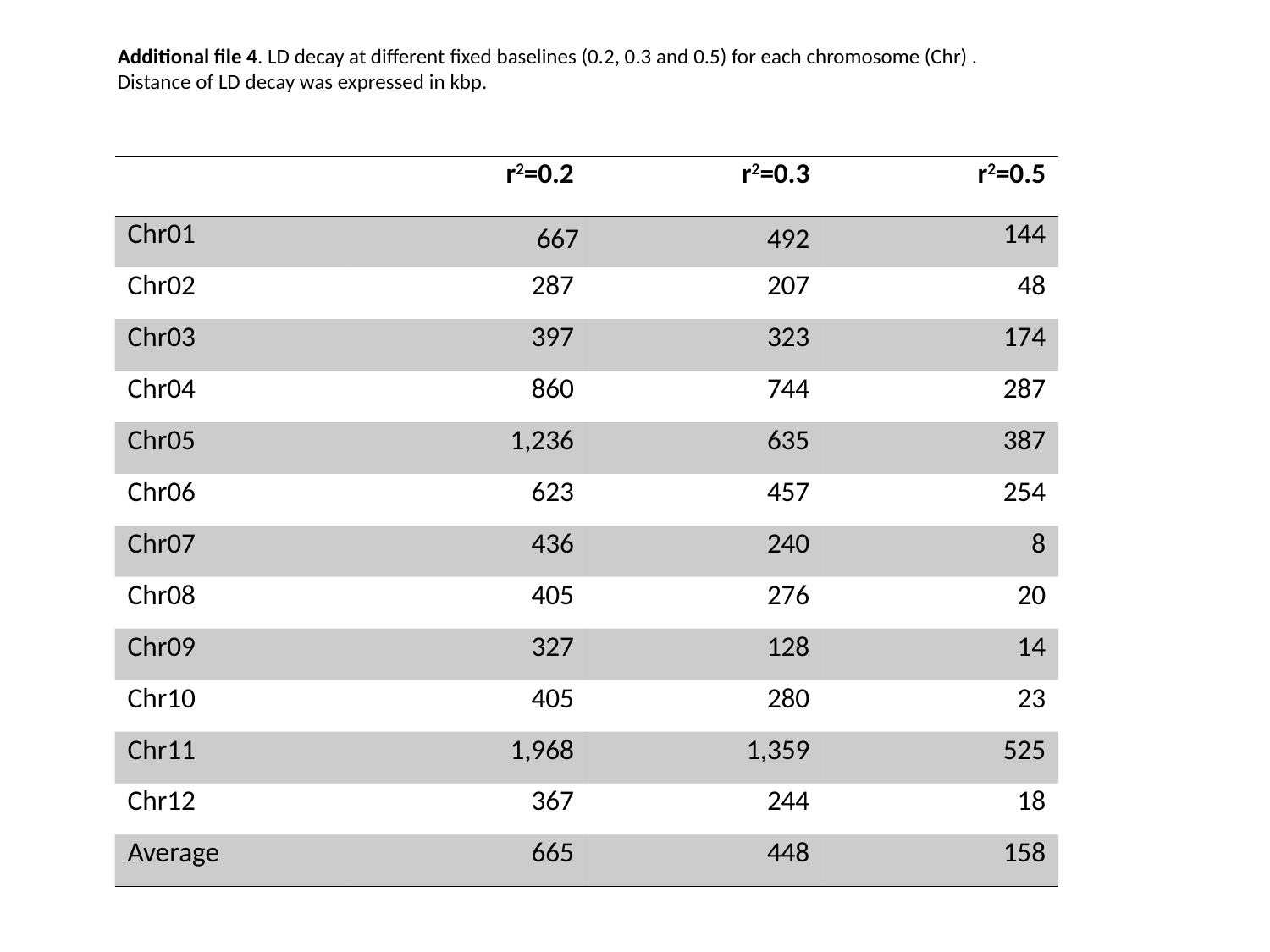

Additional file 4. LD decay at different fixed baselines (0.2, 0.3 and 0.5) for each chromosome (Chr) . Distance of LD decay was expressed in kbp.
| | r2=0.2 | r2=0.3 | r2=0.5 |
| --- | --- | --- | --- |
| Chr01 | 667 | 492 | 144 |
| Chr02 | 287 | 207 | 48 |
| Chr03 | 397 | 323 | 174 |
| Chr04 | 860 | 744 | 287 |
| Chr05 | 1,236 | 635 | 387 |
| Chr06 | 623 | 457 | 254 |
| Chr07 | 436 | 240 | 8 |
| Chr08 | 405 | 276 | 20 |
| Chr09 | 327 | 128 | 14 |
| Chr10 | 405 | 280 | 23 |
| Chr11 | 1,968 | 1,359 | 525 |
| Chr12 | 367 | 244 | 18 |
| Average | 665 | 448 | 158 |
