# Supplementary material for: An association mapping approach to identify favourable alleles for tomato fruit quality breeding
Source: BMC Plant Biol. 2014 Dec 3;14:337. doi: 10.1186/s12870-014-0337-9 (PMC4266912; doi:10.1186/s12870-014-0337-9)
Supplement: Additional file 9: — Allele effect of markers significantly associated with seven traits by Mixed Linear Model (MLM) using a MAF threshold >5%. For each marker the number of genotypes observed (Obs) and phenotypic effects (Effect) for both major and minor alleles are shown. [file 12870_2014_337_MOESM9_ESM.docx]

Additional file 9. Allele effect of markers significantly associated with seven traits by Mixed Linear Model (MLM) using a MAF threshold > 5%. For each marker number of genotypes observed (Obs) and phenotypic effects (Effect) for both major and minor alleles are shown.

|  |  | **ALLELE EFFECT^a^** | | | | | |
| --- | --- | --- | --- | --- | --- | --- | --- |
| Trait | Marker Index | Major Allele | Obs | Effect | Minor Allele | Obs | Effect |
|  |  |  |  |  |  |  |  |
| **AsA** | 2383 | A | 69 | 6.803 | G | 16 | 0 |
|  | 7588 | A | 69 | 6.803 | T | 16 | 0 |
|  | 1241 | C | 64 | 0 | A | 21 | 5.620 |
|  |  |  |  |  |  |  |  |
| **βC**^b^ | 2022 | A | 53 | 1.214 | G | 31 | 0 |
|  | 2025 | A | 52 | 1.214 | G | 32 | 0 |
|  | 2028 | A | 48 | 1.214 | G | 36 | 0 |
|  |  |  |  |  |  |  |  |
| **tLYC** | 3525 | G | 81 | 85.220 | A | 4 | 0 |
|  | 3526 | G | 81 | 85.220 | A | 4 | 0 |
|  | 3104 | G | 67 | 46.360 | A | 18 | 0 |
|  |  |  |  |  |  |  |  |
| **PHE** | 354 | A | 57 | 0 | G | 28 | 9.526 |
|  | 4365 | A | 69 | 0 | G | 16 | 12.850 |
|  |  |  |  |  |  |  |  |
| **FW** | 2992 | G | 79 | 0 | A | 6 | 96.060 |
|  | 2272 | C | 70 | 0 | A | 15 | 57.200 |
|  | 2273 | A | 70 | 0 | T | 15 | 57.200 |
|  | 2274 | G | 70 | 0 | C | 15 | 57.200 |
|  | 2275 | G | 78 | 0 | A | 7 | 77.590 |
|  | 1081 | G | 60 | 0 | A | 25 | 97.680 |
|  |  |  |  |  |  |  |  |
| **pH** | 2246 | A | 54 | 0 | C | 31 | 0.121 |
|  |  |  |  |  |  |  |  |
| **TA** | 955 | C | 80 | 0 | A | 5 | 0.196 |
|  | 2032 | A | 60 | 0 | T | 25 | 0.098 |
|  | 443 | A | 67 | 0 | T | 18 | 0.103 |
|  | 3999 | C | 69 | 0 | A | 16 | 0.103 |
|  | 1210 | A | 78 | 0 | T | 7 | 0.168 |
|  | 1010 | C | 78 | 0 | A | 7 | 0.168 |
|  |  |  |  |  |  |  |  |

^a^ The allelic effect is reported for each trait following the specific measure unit used

^b^ For βC sum of obs genotypes is 84 instead of 85 because Caro Red genotype was eliminated from analysis (see text for detailed information).
